# Prevalence of low-risk HPV types and genital warts in women born 1988/89 or 1983/84 -results of WOLVES, a population-based epidemiological study in Wolfsburg, Germany

**DOI:** 10.1186/1471-2334-12-367

**Published:** 2012-12-21

**Authors:** Karl Ulrich Petry, Alexander Luyten, Annika Justus, Angelika Iftner, Sarah Strehlke, Renate Schulze-Rath, Thomas Iftner

**Affiliations:** 1 Klinikum Wolfsburg, Frauenklinik, Schwerpunkt gynäkologische Onkologie, Sauerbruchstr. 7, 38440, Wolfsburg, Germany; 2 Conreso GmbH, Klinische Forschung, Neuhauser Straße 47, 80331, Munich, Germany; 3 Institut für Virologie, Sektion experimentelle Virologie, Universitätsklinikum Tübingen, Elfriede Aulhorn Str. 6, 72076, Tübingen, Germany; 4 Sanofi Pasteur MSD GmbH, Leimen, 69181, Germany

## Abstract

**Background:**

**Wol**fsburg HP**V E**pidemiological **S**tudy (WOLVES) is a population-based cohort study on HPV infections and associated diseases in the pre-vaccination era in young women in Wolfsburg, Germany.

**Methods:**

Women born 1983/84 or 1988/89 were invited to participate. Participants were recruited in gynecology practices, and completed a questionnaire with socioeconomic, sexual and medical data including vaccination status. Pelvic examination with Pap smear and HPV testing (HC2 = Hybrid Capture 2) was obligatory. HC2-positive and 10% of HC2-negative samples were tested for specific HPV types with SPF-10-PCR, and in inconclusive cases with DNA sequencing. Women with genital warts (GW) and those with atypical Pap smears were transferred for colposcopy. GWs were classified as typical condylomata acuminata (TCA), flat condyloma (FC) and seborrheic wart-like (SWL).

**Results:**

In total, 1258 subjects were recruited from the target population of 2850 (44.1%). Overall the prevalence of HC2 low-risk (LR) types was 8.5%. HPV6 was the most frequent LR type (2.1%), followed by HPV42 (1.1%), HPV11 and HPV44 (each 0.4%). LiPA showed a low sensitivity for HPV types 42, 90 and 91, which were detected only by HC2 and HPV sequencing. Nine women (0.7%) were transferred with incident GW: five TCA, two FC and two SWL. All TCA were associated with HPV6 in corresponding cervical swabs and warts. Tissues of SWL contained HPV6 (n = 1) and HPV16 (n = 1). The cumulative life-risk for GW was 1.4% in the 1988/89 and 4.8% in the 1983/84 cohort. Eight of 107 HC2-LR + and five of nine cases of GW had concomitant abnormal Pap smears. All CIN lesions could be linked to high-risk HPV types but borderline and low-grade abnormal smears were explained by vaginal and cervical TCA in four cases.

**Conclusions:**

HC2 was a specific test for the detection of established and potential LR types. In this first WOLVES analysis, HPV6 was the most frequent HPV type and the single LR type linked to disease. The observed GW incidence of 715 per 100,000 fits well with estimates of healthcare providers. Although life risks for GW were lower than in Scandinavian analyses, the societal burden within the WOLVES populations was considerable.

## Background

Most clinical data on the frequency of genital warts (GW) are published from clinics specialized in sexually transmitted infections (STI) or other health institutions with a focus on the treatment of diseases of the lower genital tract, while studies estimating the impact of GW on health budgets are based on data from healthcare providers [1,2]. Neither approach is optimal to measure the true prevalence and incidence of GW in the general population, nor to estimate the role of human papillomavirus (HPV) low-risk (LR) types. The frequency of GW in STI clinics depends on transfer policies and awareness among patients and transferring institutions, while the analyses of healthcare providers rely on ICD-10 codes, which may follow more economic than clinical aspects. To date, real-life population-based data on the epidemiology of LR-HPV and GW are not available for Germany. Furthermore, the term, “genital wart” is not well defined and in addition to condylomata acuminata may include mollusca contagiosa, verruca vulgaris, dermatofibroma and other skin tumors.

There is also an ongoing controversy about the best approach to measure the burden of HPV infections in the general population. In epidemiological and screening studies, Hybrid Capture 2 (HC2) showed a lower analytical sensitivity for high-risk HPV (HR-HPV) compared with different PCR-based methods. However, the clinical sensitivity of HC2 for HR-HPV-associated lesions like cervical intraepithelial neoplasia grade 3 (CIN3) and cervical cancer ranged from 93% to 100% in several controlled and randomized controlled trials. Therefore, HC2 is considered to be the best validated HPV test for cervical cancer screening [3-5]. Furthermore, HC2 is a robust test that showed almost no intralaboratory and interlaboratory variation in quality assessment trials [6]. As HC2 defines a clinically relevant HR-HPV infection it also seems useful for epidemiological studies to distinguish probably irrelevant latent from clinically meaningful HPV infections. Although this concept has been confirmed with a high level of evidence (LOE) for the detection of HR-HPV types with HC2, the clinical importance of detection of HC2-LR-HPV types has not yet been validated with a comparable LOE.

We have used data from a population-based cohort study in Wolfsburg, Germany and HC2 testing to determine the prevalence of HPV infection and associated disease. Here we report the first results on the prevalence of LR-HPV types and associated diseases. Detailed analysis of HR-HPV types will be published separately.

## Methods

### Study population

The **Wol**fsburg HP**V E**pidemiological **S**tudy (WOLVES) is a population-based cohort study on the prevalence and incidence of HPV infections and associated diseases in women of two predefined birth cohorts (born in 1984/4 or 1988/9) recruited between 19 October 2009 and 31 December 2010. The study was designed to measure the role of HPV infection in the pre-vaccination era and changes over a 5-year observation period. The residents’ registration office provided a list of all women born either 1983/84 or 1988/89 with a first residency in Wolfsburg city and 2850 women were invited by letter to attend cervical cancer screening. The invitation included information on the intention of the study and the voluntary participation. To be recruited, women had to attend one of 18 gynecologists in private practices in the city of Wolfsburg for routine Pap smear screening. Participants had to give written consent and complete a short standardized questionnaire in the private practice. Then they underwent a pelvic examination with visualization of the uterine cervix. Pap smears were taken using spatula and endocervical brush. A second sample was then obtained with a Qiagen Cervical Sampler (Medscan, Uppsala, Sweden), and suspended in 1 ml of specimen transport medium (STM/Qiagen Inc., Hilden, Germany) for HPV DNA testing.

The questionnaire included questions on education, birth country, marital status, pregnancies, parity, contraception, smoking, number of sexual partners, age at sexual debut, history of abnormal Pap smears, STI and GW. Furthermore, the referring gynecologist collected information on HPV vaccination status by checking the certificate of vaccination.

Patients were transferred for colposcopy if they suffered from GW and/or had abnormal Pap smears conspicuous of high-grade lesions or had Pap smears classified as borderline/low-grade and tested positive for high-risk (HR)-HPV.

Colposcopists classified the type of transformation zones according to the Barcelona nomenclature of the International Federation for Cervical Pathology and Colposcopy (IFCPC) [7]. In cases of type 1 or type 2 transformation zone with visible squamous columnar junction (SCJ), colposcopy was regarded as satisfactory. Any visible lesion underwent histological assessments with punch biopsies. No random punch biopsies were taken if colposcopy findings were normal. In cases of type 3 transformation zones, colposcopy with not fully visible SCJ was regarded as unsatisfactory and endocervical curettage (ECC) was obligatory. Type 3 transformation zones with visible lesions underwent punch biopsies and ECC.

**Figure 1:**
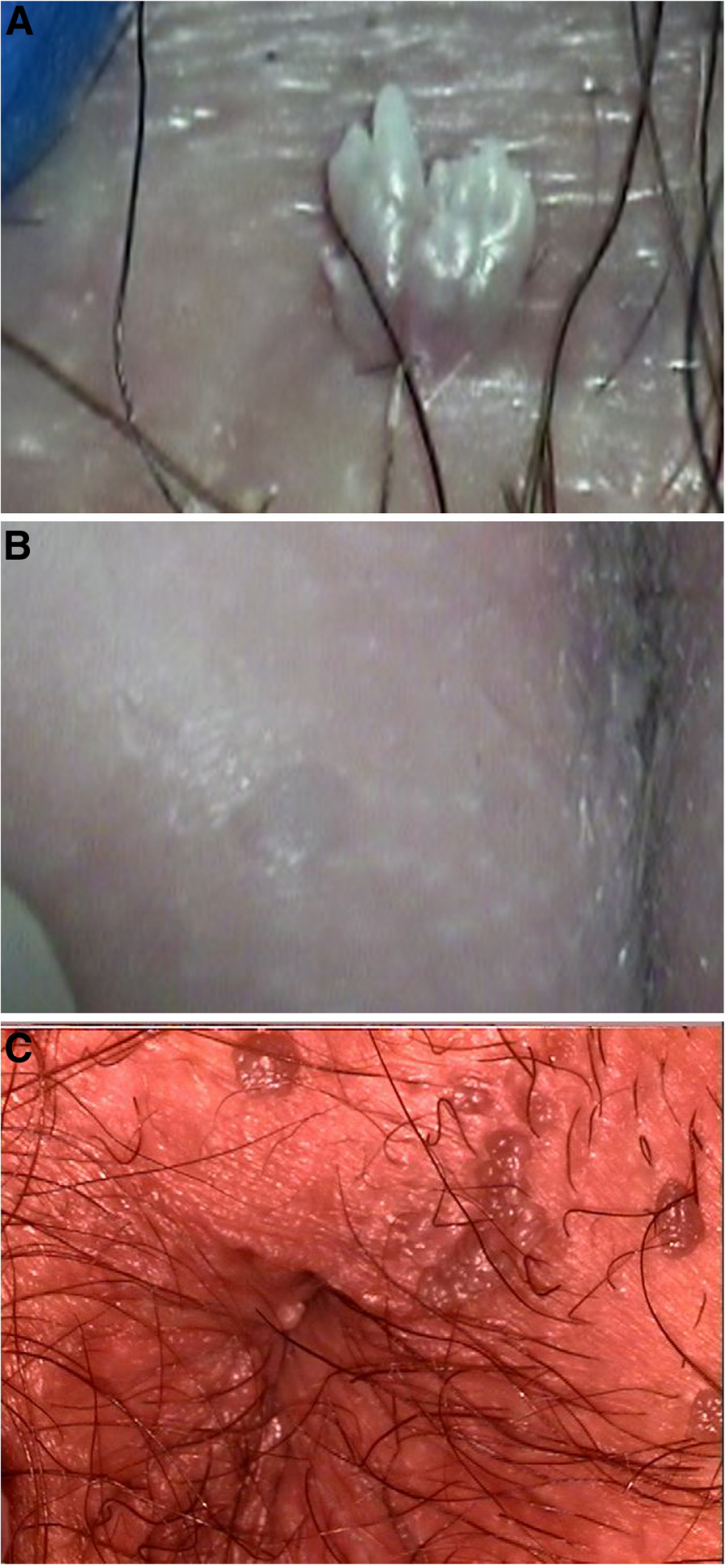
A: Typical condyloma acuminatum; B: Flat condyloma; C: Seborrheic wart like lesion.

A variety of skin and mucosal lesions may be classified as GW. Lesions were included in the category “genital warts” only if they were classified on colposcopy as:

1. “typical condylomata acuminata” (TCA) for lesions that showed the typical acuminate morphology (Figure 1A)

2. “flat condylomata” (FC) for genital papillomas with a more hyperkeratotic and papillomatous surface and flat condylomata with a smooth surface and non-pigmented papules (Figure 1B)

3. “seborrheic wart-like lesions” (SWL) of the dry skin of the external anogenital area if intraepithelial neoplasia was excluded on histology (Figure 1C).

Naevi, vulvar intraepithelial neoplasia (VIN), hirsutoidpapillomas and molluscacontagiosa were excluded.

Colposcopists and pathologists were blinded to HPV genotyping results at the time of classification of GW.

All primary HPV testing was undertaken using the HC2 assay (HC2/Qiagen Inc., Hilden Germany). All samples were analyzed for the presence of at least 13 HR-HPV types (16, 18, 31, 33, 35, 39, 45, 51, 52, 56, 58, 59 and 68) following the manufacturer’s instructions. Therefore, a positive HR-HPV result in this study refers to a patient positive for one or more of the 13 HR-HPV types included in the probe mix. Infection with LR-HPV types was also investigated by HC2. The LR probe mix included HPV types 6, 11, 42, 43 and 44. Samples were considered positive if they attained or exceeded the FDA-approved threshold of 1.0 pg HPV DNA/ml, which corresponds to 1.0 relative light unit (RLU).

All samples that tested positive for LR-HPV and/or HR-HPV with HC2 and 10% (every tenth) of all HC2-negative samples underwent HPV genotyping. HPV genotyping was performed as described previously using SPF-10-PCR, followed by Reverse Line Probe Assay LiPA Extra (SPF-10-PCR) [8]. Briefly, total DNA was isolated from the cervical samples with the use of a MagNAPure device (Roche, Indianapolis, IN) and analyzed with the use of the INNO-LiPA Extra HPV prototype assay (Innogenetics, Inc, Gent, Belgium) according to the manufacturer’s instructions. According to good laboratory practice guidelines, all PCR manipulations were performed in a laboratory that was separate from the other laboratory rooms. All PCR reactions were undertaken with 10 μl input DNA in a final volume of 50 μl using provided reagents from Innogenetics, 10 min 37°C, 9 min 94°C, and 40 cycles of 30 sec of denaturation at 94°C, followed by 45 sec of 52°C annealing temperature and 45 sec of extension at 72°C run on a MJ Thermocycler PCT 200. The PCR product was then denatured and a 10 μl aliquot hybridized to one strip at 49°C for 60 min, followed by multiple washing steps. The INNO-LiPA Extra test allowed identification of 13 established high-risk HPV types (16, 18, 31, 33, 35, 39, 45, 51, 52, 56, 58, 59 and 68), five known or putative high-risk types (26, 53, 66, 73 and 82) [9,10], seven LR-HPV types (6, 11, 40, 43, 44, 54 and 70), additional non-differentiated HPV types, and types with undefined risk (74 and 69/71) [11,12]. The strips were analyzed on a flatbed scanner with the use of LiRAS prototype software (Innogenetics, Inc), which displays the patterns and relative intensity of positive bands as arbitrary grey-tone values between 0.1 and 1.0 and allows direct data transfer to Excel spreadsheets. All HC2-LR positive samples, which were negative in the LiPA Extra, were retested with the CP4/5 PCR. The positive PCR products were purified with the Qiagen Gel Extraction Test QIAquick and then directly sequenced with the CP4 primer [13]. Sequencing allowed the identification of HPV subtypes that were not detectable by LiPA Extra.

Any lesion suspicious of high-grade neoplasia had to undergo histological assessment and patients with GW were tested for histological confirmation of the diagnosis with biopsies. Paraffin embedded tissue blocks of GW and/or cervical intraepithelial neoplasia (CIN), vulvar intraepithelial neoplasia (VIN), and vaginal intraepithelial neoplasia (VAIN) underwent HPV-genotyping with SPF-10 and LiPA. Data for the prevalence of HR-HPV, cofactors associated with the risk of HR-HPV infection, and the association between specific HR-HPV subtypes and abnormal Pap smear test results will be published separately.

The study was designed to provide a one-time cross sectional study of the 1983/84 cohort, whereas the 1988/89 cohort will be followed by annual examination for 5 years. In the year 2014/15, women born 1993/94 will be invited for a one-time examination. Overall WOLVES will allow investigation of individual changes in HPV infection during follow-up and a comparison of prevalence of HPV infections in different age cohorts over time.

WOLVES was approved by the ethics committee of the physicians’ association of Lower Saxony in Hannover, Germany (Bo/07/2009).

### Statistical analysis

All statistical analyses were undertaken by an independent statistician who did not participate in the collection of data.

The association between HPV infection and explanatory variables was analyzed in univariate (Mann–Whitney-*U* Test) and multivariate (logistic regression) analyses. Statistical analyses were performed with the validated program Testimate Version 6.5 from IDV Gauting (validation of software, hardware and user according to FDA 21 CFR Part 11). Logistic regression was calculated using SAS.

## Results

Between 19 October 2009 and 31 December 2010, 659 (43.8%) of 1504 registered women born 1983/84 and 599 (44.5%) of 1346 women born 1988/89 were recruited. The socioeconomic status of the recruited population reflected the general population in Wolfsburg.

### LR-HPV prevalence

In the 1983/84 cohort, 52/659 (7.9%) cervical samples tested positive for LR-HPV types with HC2, while in the 1988/89 cohort the corresponding prevalence was 55/599 (9.2%) (see Table 1). Within the 1983/4 cohort, 34/52 cases tested positive for HR- and LR-HPV types, while the remaining 18 patients tested positive for LR types only. The corresponding findings for the 1988/89 cohort were 32/55 and 23 cases, respectively.

**Table 1:** Age-dependent prevalence of LR-HPV (HC2) in cervical samples

	**LR-HPV positive N**	**LR-HPV positive%**	**95% CI**
1983/84	52/659	7.9	5.95 – 10.22
1988/89	55/599	9.2	6.99 – 11.78

### Co-factors of LR-HPV infection

**Figure 2:**
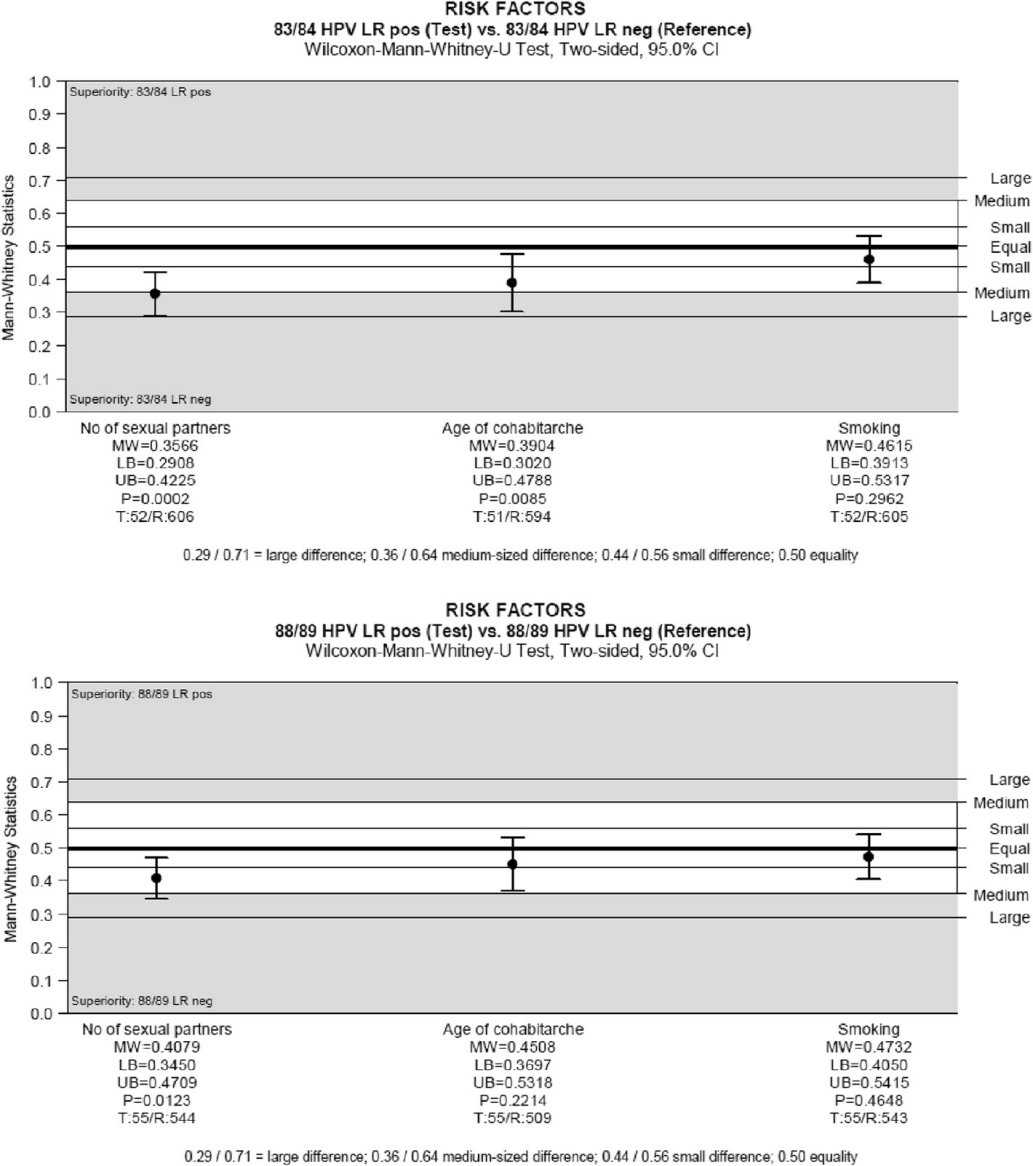
**Risk factors for LR-HPV infection. A:** 1983/84 cohort; **B:** 1988/89 cohort.

Univariate analyses with Mann–Whitney-*U* Test and multivariate analysis using logistic regression produced identical results. Univariate analyses showed no association between risk for HC2-LR and smoking, education, contraception, country of origin, age at first menstruation and history of STI. A higher number of sexual partners was associated with a significantly increased risk for detection of HC2-LR types in both age cohorts, but this effect was more pronounced in the 1983/84 cohort. Young age at first intercourse was found to be a weak risk factor for HC2-LR, but only in the 1983/84 cohort (Figure 2). Multivariate analysis identified only the number of sexual partners as significant risk factor for HC2 (Table 2). However, as this is not a confirmative study, these results should be interpreted only descriptively.

**Table 2:** Multivariate analysis using logistic regression

	**Odds ratio**	**95% CI**	**Odds ratio**	**95% CI**
	**2–5 vs 1 partner**		**>5 vs 1 partner**	
1983/84	9.8	3.1 – 28.0	21.5	7.1 – 61.4
1988/89	2.9	1.6 – 5.4	6.2	3.0 – 12.8

Number of lifetime sexual partners was the single significant risk factor for HPV infection.

Restricting the analyses to women positive only for LR-HPV types showed no difference in risk factors between LR-HPV only and LR-/HR-HPV (data not shown).

### HPV genotyping

Two of 107 HC2-LR positive samples failed in HPV genotyping with SPF-10-PCR. LR-HPV genotypes 6, 11, 40, 42, 43, 44, 54, 61, 70, 72 and 81, as defined by Munoz [10], were detected in 38 cases. HPV types 69, 71 and 74, which are closely related to LR types and are not associated with cancer [11], were detected in another five cases. HPV types 53 and 66, which are listed as putative HR types by IARC [12], were detected in another 13 cases. The genotype remained unknown in another six PCR-positive cases.

Based on SPF-10-PCR genotyping of HC2-LR positive cases, HPV6 was the most frequent LR type (n = 26), followed by HPV11 (n = 5), HPV44 (n = 5), HPV43 (n = 2), HPV54 (n = 1) and HPV70 (n = 1).

**Figure 3:**
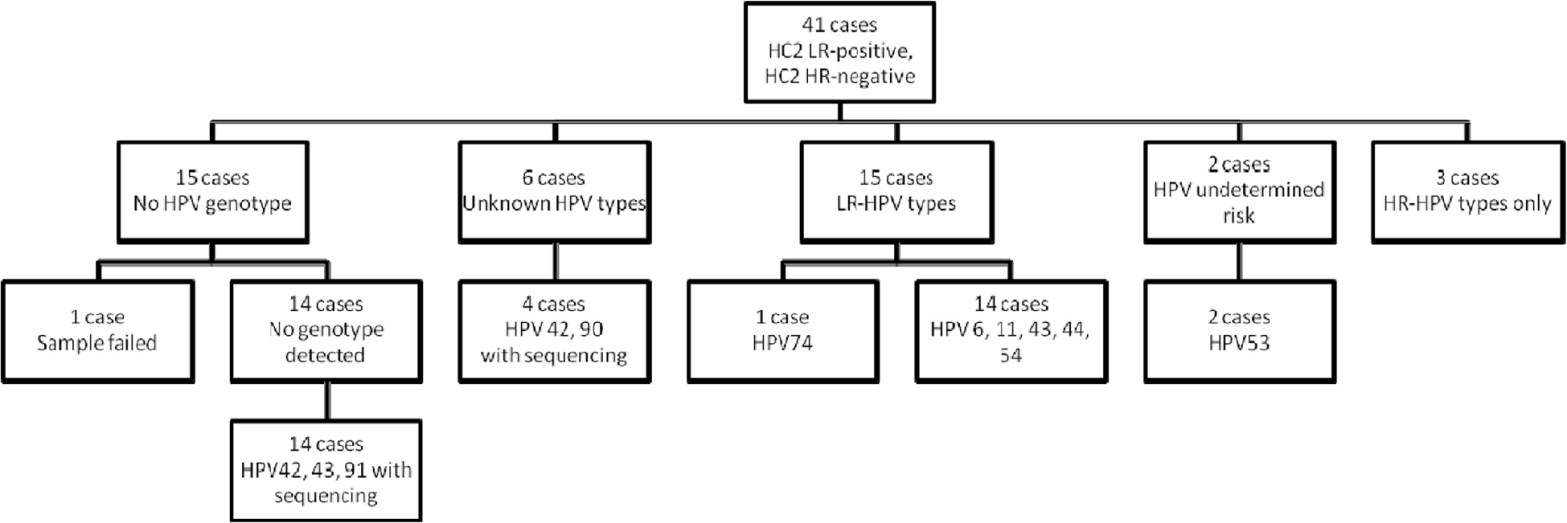
Genotyping results with LiPA for all 41 cases that tested positive for LR-HPV only with HC2 and HPV-DNA sequencing of LiPA-negative/HC2-LR-positive cases.

**Figure 4:**
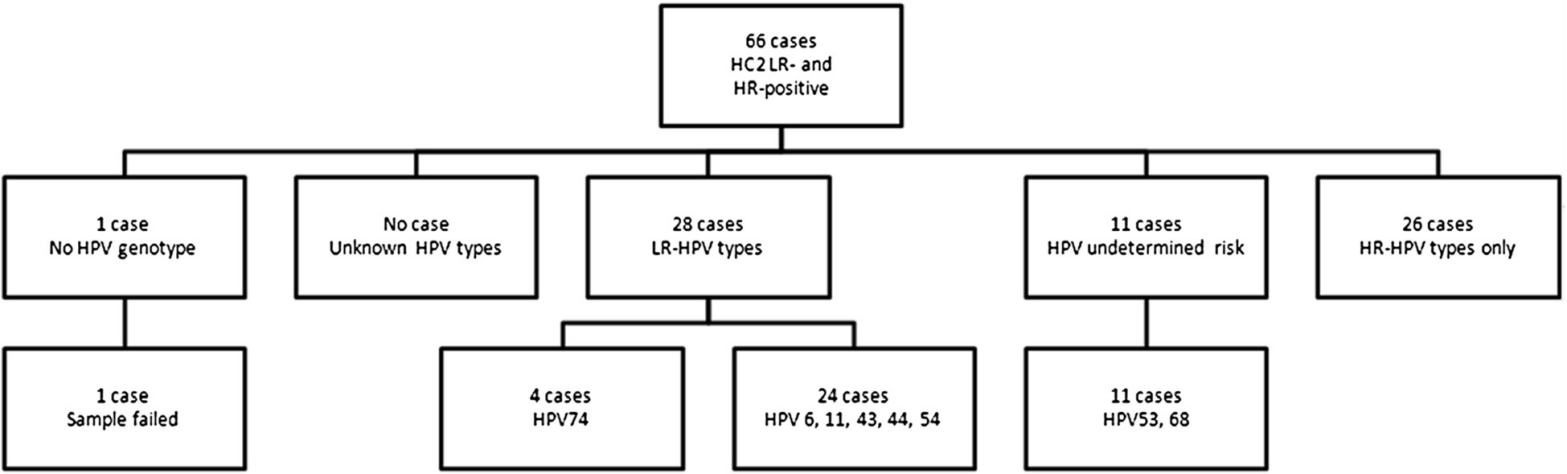
**Genotyping results with LiPA of all 66 cases that tested positive for LR- and HR-HPV with HC 2.** HR-types included: HPV types 16, 18, 31, 33, 35, 38, 45, 51, 52, 56, 58, 59 and 68.

In 43/105 samples, genotyping showed either no HPV types at all or only HR types (Figures 3 and 4). In 29 HC2-LR-positive samples, genotyping detected only HR-HPV types; LiPA detected only HR-HPV in 26 cases that tested positive for LR-HPV (± HR types) with HC2. PCR and LiPA showed negative results in another 14 cases. Unknown HPV types and negative PCR results were observed exclusively in HC2-LR-positive cases that tested negative for HC2-HR.

Cases with negative PCR, but positive HC2-LR, did not differ in HC2-LR signal strength from other HC2-LR positive samples. The mean signal strength was 155.43 RLU with a range from 1.05 to 1,021.33 RLU. All 14 cases with HC2-LR-positive but SPF-10-PCR-negative results and all six HC2-LR-positive cases with unknown genotypes underwent further evaluation by CP4/5 PCR and direct DNA sequencing of the amplification product. Among these 20 cases, one sample failed, HPV42 was detected in 13 samples, HPV91 in three cases, while HPV90, HPV43 and HPV45 were each found in one of the remaining cases.

Eighty-nine samples that tested negative for LR and HR types with HC2 were randomly selected for genotyping. LiPA detected two cases of LR-HPV infection as defined by IARC (one HPV44 and one HPV54) and two cases associated with HPV74.

### Genital warts

Thirty women had a history of GW, 26/659 (3.9%) in the 1983/84 cohort and 4/599 (0.7%) in the 1988/89 cohort. Among cases with GW prior to the start of WOLVES, 22 underwent pharmaceutical treatment and eight were treated surgically. Another five women born 1983/84 and four born 1988/89 were transferred to colposcopy because of first-time GW.

The colposcopy classification was TCA in five cases, FC in two cases and SWL in two cases. Both cases of SWL and one FC tested negative for HC2-LR, while all five cases of TCA and one FC were associated with cervical HC2 samples that tested positive for LR-HPV. Genotyping detected HPV6 in all five cervical samples of patients with TCA either as single HPV or as one of multiple HPV types. In two TCA cases, biopsies were taken and PCR genotyping confirmed HPV6 in the tissues. The remaining patients with TCA and FC refused biopsies. Both SWL underwent histological assessment. One case of warts close to the pubic mount was associated with HPV6, while the second case of SWL of the greater lips of pudendum contained HPV16.

All patients with TCA and SWL were smokers and not HPV vaccinated. One patient with FC of the perineum was a smoker and received a full course of quadrivalent HPV vaccination 3 years prior to diagnosis. The cervical sample tested negative for HC2-LR types. The remaining patient with FC never smoked and was not vaccinated. The cervical sample tested positive for HR- and LR-HPV types. Genotyping showed HPV types 51, 52 and 66.

### Abnormal smears and colposcopy findings

Overall 8/107 LR-HPV-positive women showed abnormal Pap smears, including five cases of low-grade squamous intraepithelial lesion (LSIL) and three cases with borderline cytology. All occurred exclusively in women who tested positive for HC2 LR and HR, while no case was observed in HC2-LR-positive/HC2-HR-negative participants.

Five of nine patients with GW had abnormal Pap smears classified as borderline (n = 1), low-grade squamous intraepithelial lesion (n = 3) and high-grade squamous intraepithelial lesion in one patient with SWL whose cervical sample tested positive for HR-HPV, but negative for LR-HPV types.

The colposcopic evaluation of all five TCA cases excluded any cervical abnormality in three women with normal or borderline Pap smears. The remaining two cases had Pap smears classified as LSIL. One case of TCA was diagnosed with a CIN1 lesion. PCR genotyping of the CIN1 tissue detected HPV51 as single HPV type. The remaining TCA cases had cervical condylomata acuminata, the tissue contained HPV6 as single HPV type.

## Discussion

WOLVES has provided the first real-life data on the epidemiology of LR-HPV and associated diseases in Germany. Of particular interest is the observation that the prevalence of LR-HPV is declining in the 1983/84 cohort, suggesting that spontaneous regression is common. Furthermore, the results from this analysis also suggest that the number of sexual partners is the most important risk factor for LR-HPV infection. The reported HC2-LR prevalence of 7.9–9.2% is concordant with the 8.5% prevalence in 25–34 year-old Italian women observed by Rossi and coworkers [14]. The Italian trial found HPV types 6 and 11 in only 19.9% of all HC2-LR-positive samples compared with 27.6% (29/105) in the WOLVES population. However, genotyping could not detect any HPV type in 14 HC2-LR-positive samples and found only HR types as defined by IARC in another 29 samples, 26 of which tested positive for HC2-HR and -LR. Using additional data from CP4/5 PCR and direct HPV-DNA sequencing, 18 of 20 HC2-LR-positive cases with either PCR-negative results or unknown HPV genotypes were classified as HPV infections with types 42, 43, 45, 90 and 91. HPV-genotyping using SPF-10-PCR is known to have a very high analytical sensitivity for most HPV types but obviously this system has a lower sensitivity to detect HPV42, as described previously [8], although this HPV type was the second most common LR-type in the WOLVES study population. Because of this weakness, the observed overall sensitivity of SPF-10-PCR for LR-HPV was lower than for HC2. Based on SPF-10-PCR genotyping, HPV6 was the most frequently found LR-HPV type. HPV42 and, to a lesser extent, HPV90 and HPV91 infections probably account for most cases classified as HC2-LR- and HC2-HR-positive, SPF-10-PCR–LR-negative; therefore, we conclude that the majority of HC2-LR-positive results are explained by infections with LR-types, as defined by Munoz et al., while the remaining HC2-LR-positive cases can be attributed almost entirely to infections with other probable LR types (HPV types 74, 90 and 91) and HPV types with undetermined risk (HPV types 53 and 66). We found almost no signs of HC2-LR cross-hybridization with HR-types. The detection of HR-HPV types with SPF-10-PCR in HC2-HR-negative women and identification of LR-HPV by SPF-10-PCR in few HC2-negative controls is most likely explained by latent or low replication infections. The finding that HC2-LR identifies HPV types 6, 11, 42, 43 and 44 that are included in the probe mix and all related HPV types with LR properties is probably attributable to the use of an RNA probe hybridizing to the full genome. The single “low-risk type”, according to Munoz et al., that was missed by HC2-LR was HPV70; in the WOLVES study, all six cases tested positive for HC2-HR. However, it has been shown that HPV70 immortalizes primary keratinocytes [15]. A proposal for a new classification of HPVs by Bernard et al. concluded that HPV70 is a HR type of species 7, closely related to HPV18 and HPV74, a LR type of species 10 closely related to HPV types 6, 11 and 44 [16]. Both proposals are in agreement with our findings.

HPV6 was the most frequent of all identified LR-HPV types (26 cases) and, to date, the single LR type associated with clinical disease in our study population. One focus of WOLVES is a careful colposcopic distinction between HPV-related lesions in groups with different specific morphologies. We hypothesize that TCA are linked exclusively to HPV6 and HPV11, whereas the approximately 10–17% of GW associated with other HPV types observed in a number of different studies may be explained by FC and SWL [17-19].

In the WOLVES study population, the majority, but not all, external GWs were confirmed as TCA. All five cases with typical acuminate warts were associated with HC2-LR-positive cervical samples and detection of HPV6 by SPF-10-PCR genotyping, while cervical samples of both cases with SWL lesions tested negative for HC2-LR, although HPV6 and HPV16 were detected in the wart tissue. The number of GW classified colposcopically is still too small for an assessment of our hypothesis of HPV type-specific clinical pictures, but we hope that follow-up data over 5 years from WOLVES and other studies will provide definitive evidence.

The observed incidence of 715 GW per 100,000 women (9/1258) during the observation period corresponds well with calculations based on healthcare providers’ studies and cross-sectional studies for urban regions in Western Germany and shows that these approaches may provide reliable data [1,20]. In the 1983/84 cohort, 3.9% (26/659) reported a history of GW compared with 0.7% in the 1988/89 cohort; the corresponding total life risks for GW on 31 December 2010 were 4.8% (1983/84) and 1.4% (1988/89). If the younger population (1988/89 cohort) continues with similar incidence rates over the next 5 years, the cumulative rate of GW will be lower than the corresponding rate observed in the 1983/84 cohort. The most important explanation for this emerging pattern may be that 126 women born 1988/89 (21%) had a full course of quadrivalent HPV vaccination compared with only 40 women (6%) in the older cohort. It has been shown that HPV vaccination is highly efficient in preventing GW and will result in a rapid decline of this disease within a few years in populations with high vaccine coverage [21,22]. After correction for this potential bias, by exclusion of vaccinated participants in our study population, both cohorts are likely to show similar total life risks at the same age in 2010 and 2015.

Although we found a significant burden of disease, neither incidence nor life-time risk for GW reached the high levels observed in four Nordic countries [23]. However, in Wolfsburg, as seen in other investigations, GW were associated with a need for multiple treatments and had an adverse impact on psychological, social and sexual functions [24]. Our real-life findings agree with cross-sectional investigations showing that the estimated costs associated with the treatment of GW is substantial [20,25].

The results of the multivariate logistic regression showed that only a higher number of sexual partners increased the risk for LR-HPV type infection. Other risk factors such as age at sexual debut or smoking were not significant. Nevertheless, it is notable that eight of nine patients with GW were current smokers. Although the numbers are too small for statistical analysis, our observation is consistent with a concept that risk factors for the acquisition of HPV infection are different from those (co-factors) associated with the development of clinically relevant disease. The number of sexual partners and smoking have been identified as risk factors associated with HPV infection in other recent studies, although these data related to overall prevalence of HPV rather than LR-HPV specifically [26,27].

LR-HPV types are purported to cause a substantial proportion of low-grade and borderline findings on cytology. We also found an increased rate of abnormal Pap smears (8/107), but most cases were related to HR-HPV types in women with multiple HPV-type infections. Three out of five patients with TCA had abnormal Pap smears and two of these abnormalities were linked to vaginal and/or cervical condylomata.

## Conclusions

WOLVES is the first population-based trial that provides real-life data on the epidemiology of LR-HPV in young women in Germany. Our standardized approach to investigate the epidemiology of LR-HPV types in young German women with the use of HC2 seems feasible. Genotyping of an HC2-negative control group shows that a small number of probably latent infections will be missed by this concept, but HC2-LR accurately detected all LR-HPV types, defined by Munoz et al., and related types (e.g. HPV74) that were described in recent studies as LR-HPV. We speculate that HC2-LR may be useful in discriminating future LR from HR-HPV types. The observed LR-HPV prevalence of 8.5% is in accordance with results from similar studies in Italy and Spain [14,28].

HPV6 was the most common LR type found in the WOLVES population, followed by HPV types 42, 11 and 44. In contrast, the prevalence of HPV6 was only 2.1% and less than one-quarter of HC2-LR cases were related to HPV6, while all cases of LR-HPV-associated diseases were exclusively related to this type. Of 26 cases with cervical HPV6 infections, five presented with TCA, one with FC and three showed abnormal Pap smears. We expect to diagnose lesions related to other LR-HPV types during future follow-up, but in accordance with other investigations our findings underline the peculiar role of HPV6 and question the clinical relevance of LR-HPV infections other than HPV types 6 and 11.

Most established co-factors in the genesis of HR-HPV infections and associated neoplasia and GW showed no association with LR-HPV infections in the WOLVES study population. The single co-factor associated with a significantly increased risk for LR-HPV was a high number of sexual partners. This may be different for the development of GW, which were observed almost exclusively in smokers, and further research is mandatory. Similarly, our hypothesis of an HPV type-specific clinical morphology of lesions needs to be confirmed in larger patient numbers. To date, all TCA were related to HPV6.

## Competing interests

KU Petry received speaker’s honorarium from Beckton Dickinson, Qiagen and Roche Diagn. R Schulze-Rath is employee of Sanofi Pasteur MSD. T Iftner has received institutional grants from GSK, Gen-Probe, Sanofi-Pasteur MSD and Hologic. The remaining authors declare no conflict of interest.

## Authors’ contributions

KUP was responsible for the overall study design, data analysis, interpretation, writing of the manuscript and data collection. AL was responsible for data collection and interpretation. AJ was responsible for statistical analyses and interpretation. AI was responsible for HC2-testing, HPV genotyping and sequencing, data collection and interpretation. SS was responsible for trial coordination, quality control and data collection. RSR was responsible for trial coordination and interpretation. TI was responsible for the study design, HPV testing, data collection and interpretation. All authors read and approved the final manuscript.

## Pre-publication history

The pre-publication history for this paper can be accessed here:

http://www.biomedcentral.com/1471-2334/12/367/prepub
